# Development of a newly immunoassay specific for mouse presepsin (sCD14-ST)

**DOI:** 10.1038/s41598-022-22096-1

**Published:** 2022-12-15

**Authors:** Gaku Takahashi, Kouichi Hoshikawa, Rioto Suzuki, Kotaro Sato, Shintaro Hoshi, Daisuke Yoshinao, Kamon Shirakawa

**Affiliations:** 1 Department of Critical Care, Disaster and General Medicine, School of Medicine, Iwate Medical University, 2-1-1 Idaidori Yahaba Town, Iwate, 028-3695 Japan; 2 Clinical Development Group, LSI Medience Corporation, Tokyo, Japan

**Keywords:** Biomarkers, Molecular medicine

## Abstract

Presepsin (sCD14-ST) is used as a marker for sepsis diagnosis. The production mechanism of presepsin is unique in that it is produced through phagocytosis of microorganisms. However, some studies have demonstrated that non-infected patients had increased presepsin levels and that presepsin is related to the risk or severity of diseases. This study was designed to describe a sensitive sandwich enzyme-linked immunosorbent assay for mouse presepsin developed to investigate the association of presepsin with diseases. Polyclonal antibodies were generated from peptide-immunized rabbit antiserum. Mouse presepsin standard was prepared using the recombinant method as an Fc-fusion protein. The linear detection range of the method was 4.7–300 pg/mL with a detection limit of 1.4 pg/mL. The assay detected mouse presepsin where mouse soluble CD14 (sCD14) was digested by cathepsin D proteinase and the cross-reactivity of sCD14 was not observed. The normal levels of mouse presepsin and sCD14 were compared; 65.9 ± 21.4 pg/mL and 43.2 ± 7.2 ng/mL were determined, respectively. Moreover, the levels of presepsin and sCD14 were compared with a lipopolysaccharide (LPS)-injected sepsis mouse model. The newly developed analytical method had high specificity to presepsin and is an efficient tool for studying the association between presepsin and diseases.

## Introduction

Presepsin (sCD14-ST) is a fragment of CD14, a receptor for lipopolysaccharide (LPS), which is a glycoprotein of the N-terminal of CD14 (amino acid sequences from 1 to 64), and its molecular weight is 13 kDa^1^. Yaegashi et al.^2^ demonstrated that presepsin increased in septic patients and discriminated sepsis from systematic inflammatory response syndrome (SIRS). Subsequently, the chemiluminescent enzyme immunoassay kit was developed, and a diagnostic biomarker for sepsis was launched^3^. Now, its usefulness in diagnosing sepsis has been widely studied. Arai et al. have reported that presepsin was produced by cleavage of CD14 through enzymes associated with phagocytosis by monocytes and neutrophils. In addition, they have demonstrated that soluble CD14 (sCD14) is cleaved by elastase and produces presepsin in vitro^4^. In rabbit sepsis models, phagocytosis was needed to produce presepsin and cathepsin D, a lysosomal enzyme that cleaves sCD14 and produces presepsin with a molecular weight of 13 kDa^5^. On the contrary, some studies have demonstrated that presepsin elevation is not through phagocytosis. Chenevier-Gobeaux et al.^6^ have reported that presepsin is produced by the monocytic THP-1 cell line when LPS stimulates THP-1 cells. Furthermore, the presepsin levels increase in patients with systemic lupus erythematosus (SLE) who are non-infected and are related to the risk or severity of diseases, in which its production mechanism and relation to diseases are unknown^7^.

Therefore, to examine the relationship between presepsin production and diseases, we developed polyclonal antibodies specific to mouse presepsin and evaluated the ability of these antibodies to recognize mouse presepsin. Furthermore, we developed a mouse presepsin-specific enzyme-linked immunosorbent assay (ELISA) using two anti-mouse presepsin antibodies that recognize mouse presepsin, but not sCD14, in the blood. Using the newly developed immunoassay, we could determine the plasma levels of presepsin and compared with the levels of sCD14 in healthy mice and LPS-injected sepsis mouse model that displayed the SIRS features of sepsis.

## Results

### Characterization of C-pep8 and N-pep2 polyclonal antibodies using ELISA

**Figure 1:**
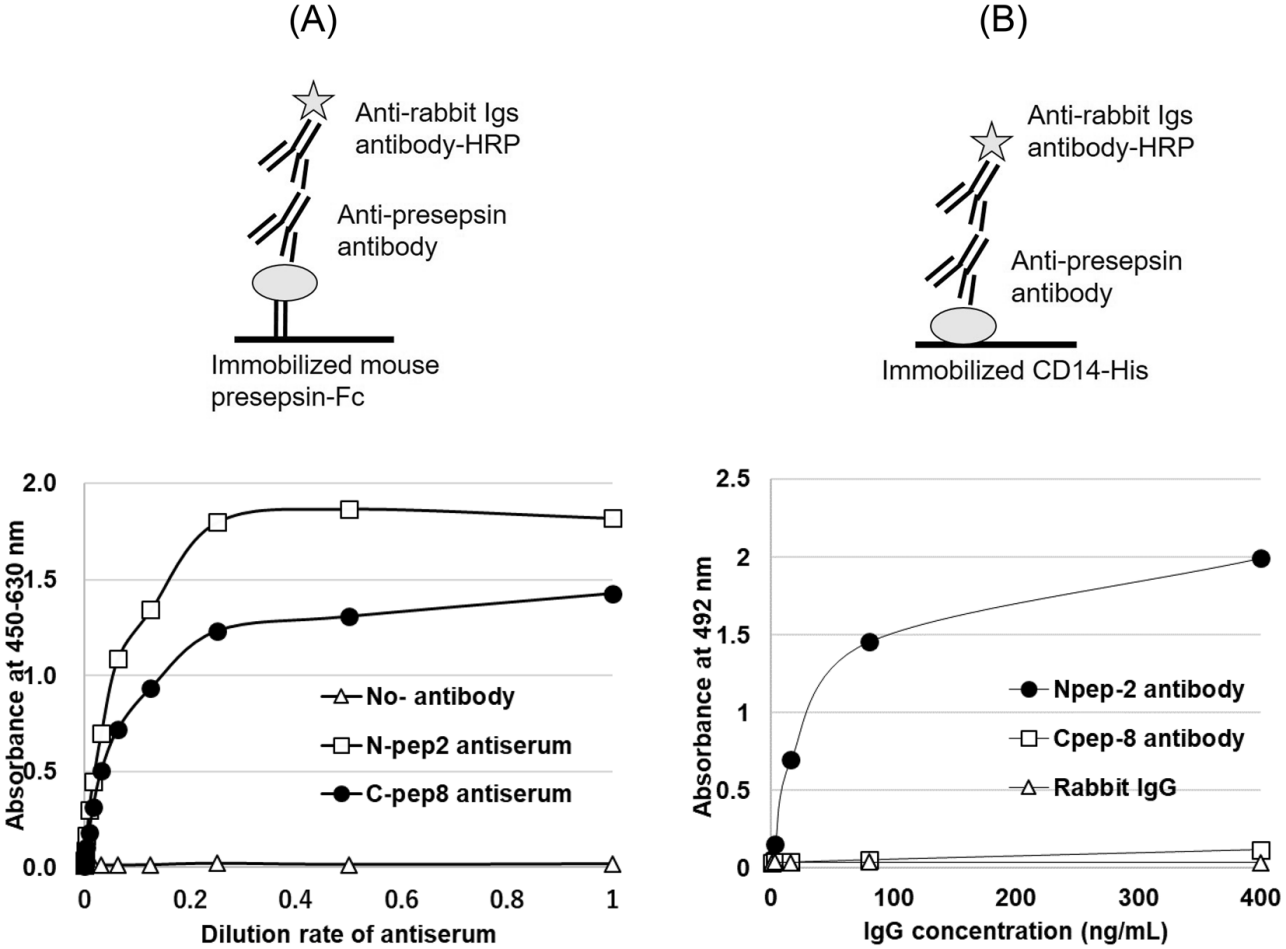
Specificity of the established polyclonal antibodies (N-pep2 and C-pep8) for mouse presepsin as assessed by immobilized antigens. (**A**) Sequential diluted polyclonal antibodies (culture supernatant) were incubated with immobilized recombinant mouse presepsin-Fc (sCD14-ST-Fc) and binding antibodies were detected by peroxidase-labeled anti-rabbit immunoglobulins antibody (DAKO, P0448). Binding activity is shown by the absorbance of 450–630 nm. (**B**) Diluted purified polyclonal antibodies were incubated with immobilized recombinant mouse CD14-His and binding antibodies were detected by peroxidase-labeled anti-rabbit immunoglobulins antibody (DAKO, P0448). Binding activity is shown by the absorbance of 492 nm.

To characterize the established polyclonal antibodies, namely, C-pep8 and N-pep2, reactivity to sCD14-ST-Fc was examined using an antigen-immobilized ELISA. Anti-C-pep8 and anti-N-pep2 antibodies can bind to sCD14-ST-Fc (mouse presepsin) (Fig. 1A). The binding-activity of each antibody was examined using sCD14-His antigen-immobilized ELISA. In the antigen-immobilized ELISA, the addition of increasing amounts of C-pep8 antibody had no binding-activity to sCD14-His; however, the N-pep2 antibody had binding-activity to sCD14-His (Fig. 1B).

### Development of a sandwich ELISA for mouse presepsin

To develop a newly sandwich ELISA for determining the concentration of mouse presepsin, the C-pep8 (capture antibody) and biotinylated-N-pep2 (detection antibody) antibodies were used. The lower detection limit was determined to be 1.4 pg/mL as the baseline + 2 standard deviation defined by the measuring standard dilution buffer (n = 24).

### Validation of the newly developed ELISA for quantifying presepsin in mouse plasma

**Figure 2:**
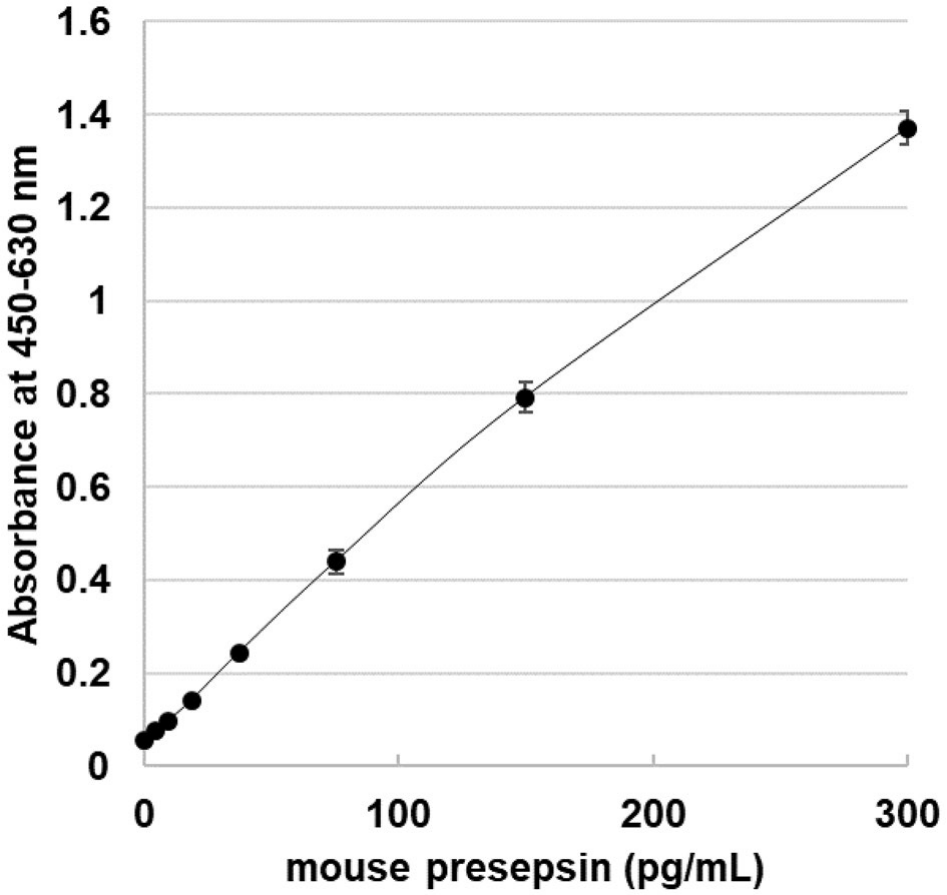
Representative mouse presepsin standard curve with dynamic range 4.7–300 pg/mL of presepsin-Fc protein of the developed ELISA. Each point representative of duplicate measurements.

**Figure 3:**
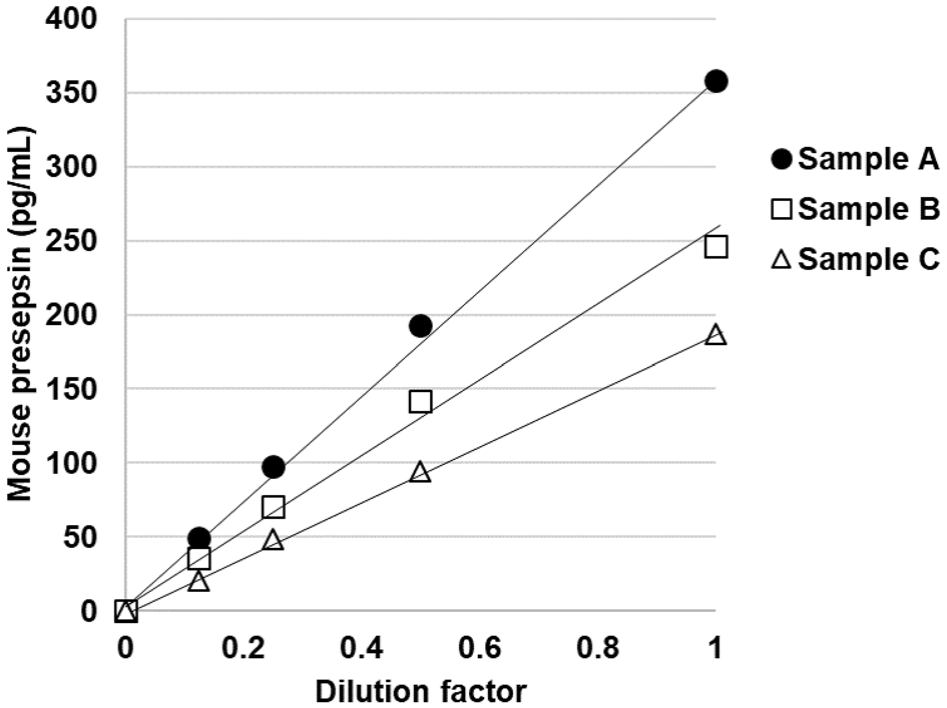
Linearity of the developed ELISA for mouse presepsin in serial diluted mouse serum. The three individual serum samples were serially diluted as indicated for determination by the developed ELISA. Each point representative the mean value of duplicate measurements.

The standard curve of mouse presepsin had good dilutional linearity in the range of 4.7–300 pg/mL (Fig. 2). The intra-assay CV (n = 10) was 2.7–3.5% for mouse presepsin. Moreover, we evaluated the dilutional linearity of measuring the plasma levels of presepsin using the newly developed ELISA. The measurement of the twofold serial dilutions (1/2, 1/4, 1/8, and 1/16, v/v) of three mice serum samples exhibited good linearity from 1/2 to 1/16 dilution (Fig. 3), but 1/2 dilution was needed for accurate determination. The results indicated that no factors in healthy mice interfered with the newly developed ELISA as long as serum was used at 50% or less.

The specificity of the newly developed ELISA was determined using recombinant CD14-His and purified mouse rsCD14ST (presepsin)-Fc. The cross-reactivity of mouse CD14 was less than 0.001% at the maximum concentration (200 ng/mL is 667 times higher of the concentration of near the upper limit of quantitation of mouse presepsin) that can be added (original data is presented in Supplementary Fig. 6).

To further evaluate the analytical recovery of mouse presepsin that spiked into mouse serum, the lowest-dose sample was selected and spiked with mouse presepsin at the indicated concentration (i.e., 10, 20, 50, 100, 200, or 500 pg/mL). The spiked samples were measured using the newly developed ELISA. The recovery ratio at all three concentrations ranged from 89.8% to 97.6%, indicating that the assay has good accuracy in the range of 4.7–300 pg/mL even in the presence of 50% mouse serum (Table 1).

**Table 1:** Recovery of mouse presepsin from six different spiked concentrations.

Spiked value (pg/mL)	Recovery value (pg/ml)	Recovery (%)
10	9.3	93.0
20	18.3	91.5
50	44.9	89.8
100	97.6	97.6
200	191.3	95.7
500	477.8	95.6

### Reactivity of CD14-His and cathepsin D-digested CD14-His measured using the newly developed ELISA

**Figure 4:**
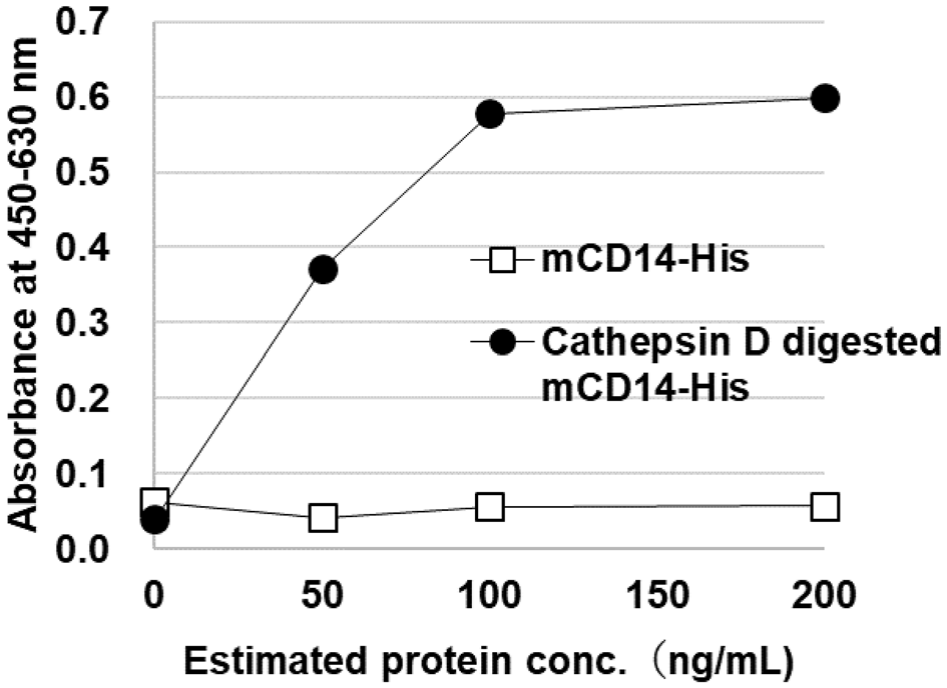
Reactivity with recombinant mouse CD14-His and mouse presepsin prepared from mouse CD14-His by incubation with cathepsin D. Two antigens were assessed using the developed ELISA. Absorbance is shown by the mean value of duplicate measurements.

No reactivity with mouse CD14-His was observed using the newly developed ELISA. However, reactivity with cathepsin D-digested mouse CD14-His was observed (Fig. 4). The digestion of mouse CD14-His was confirmed by Western blotting using the anti-His antibody (MBL, Tokyo, Japan) and anti-presepsin antibody (anti-N-pep2). The molecular weight of mouse CD14-His (approximately 50 kDa) was detected using the anti-His antibody, and the digested mouse CD14-His fragments were detected by anti-presepsin antibody (anti-N-pep2) (original blots are presented in Supplementary Fig. 7A,B).

### Determination of intact presepsin in mouse plasma using the newly developed ELISA and comparison with the sCD14 level

The plasma levels of presepsin and sCD14 were compared between the healthy (n = 20) and LPS-injected sepsis mouse models (n = 20). The level of mouse presepsin was 65.9 ± 21.4 pg/mL in the healthy mice and 29.8 ± 14.5 pg/mL in the sepsis model; thus, the sepsis group had a significantly lower presepsin level than the healthy mice (*p* < 0.001). Moreover, the sCD14 levels were 43.2 ± 7.2 and 186.3 ± 20.4 ng/mL in the healthy mice and sepsis model, respectively; thus, the sepsis model had a significantly higher sCD14 level than the healthy mice (*p* < 0.001).

## Discussion

The determination of the circulating levels of presepsin has been shown to be an attractive strategy for monitoring the degree of severity of sepsis or other diseases^8–10^. In this study, we established a novel polyclonal antibody (C-pep8) specific for the digested CD14 fragment—presepsin (sCD14-ST)—and developed a sandwich ELISA for determining presepsin in mouse plasma with high sensitivity.

**Figure 5:**
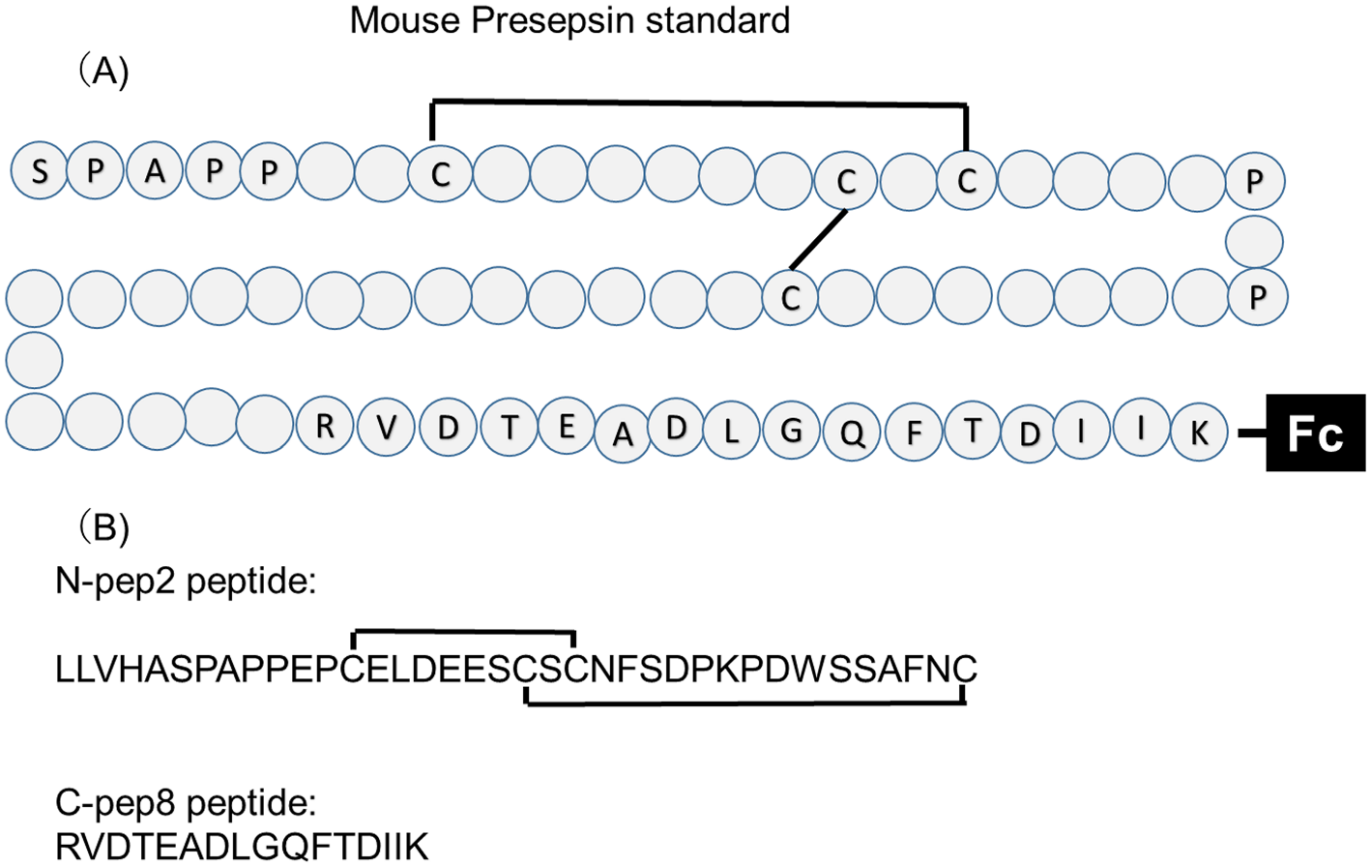
Structure of mouse presepsin and its amino acid sequences in CD14. (**A**) Presepsin standard is a 68-residue-long single polypeptide having two intramolecular disulfide bonds. CD14 estimated to processed by proteolytic cleavage at the cathepsin D cleavage site (64R–65E) to generate presepsin (sCD14-ST) in mice. (**B**) Amino acid sequences of immunized peptide for generate anti-mouse presepsin antibodies.

CD14 comprises primary proteolytic cleavage to generate presepsin (amino acid sequences from 1 to 64 a.a.)^1,3,4^. Juan et al. have reported that the LPS-binding site of CD14 is an amino acid of 7–10 and 57–64 a.a.^11^. However, they have also reported that the minimum unit of sCD14 for binding LPS is 152 a.a., indicating that the LPS-binding ability depends on the length of CD14^12^. Verelst et al. have examined the diagnostic potential of monoclonal antibody (mAb), the conformation ability, of which appeared to depend on the specificity to Tau insert, and found that the mAb can discriminate patients with Alzheimer’s disease from healthy individuals^13^. In addition, Usami et al. have developed a monoclonal antibody that specifically recognizes C-terminally truncated ApoA-I by immunizing mice with a peptide that selectively reacts to recombinant C-terminally truncated ApoA-I but not recombinant full-length ApoA-I^14^. Hence, we hypothesized that the polyclonal antibody specific for the novel generated C-terminal is the most important component of the ELISA for the measurement of mouse presepsin (Fig. 5). We synthesized the linear 16-mer peptide of the homolog of human sequence for preparing a mouse presepsin-specific antibody as an immunogen. The 16-mer peptide raised a high titer of antisera against the immunogen peptide in two rabbits, which reacted to the sCD14-ST-Fc antigen. We confirmed that the C-pep8 antibody reacted to the immunogen and mouse sCD14-ST-Fc but not mouse CD14-His (Fig. 1A,B). These results indicated that the developed anti-mouse presepsin polyclonal antibody C-pep8 was specific for mouse presepsin. Among the other antibodies used to generate a sandwich ELISA, we selected the 37-mer peptide, which has two sulfhydryl bonds similar to that in the intact CD14. The antisera against the immunogen reacted not only to sCD14-ST-Fc but also to sCD14 (Fig. 1A,B).

We selected a two-step sandwich ELISA and immobilized the specific antibody (C-pep8). First, the immobilized C-pep8 antibody bound to presepsin only; subsequently, the plate was washed, and excess sCD14 was removed from the reaction. The nonspecific N-pep2 antibody bound to the C-pep8 antibody–presepsin immune-complex. The newly developed ELISA demonstrated a range of standard curves for mouse presepsin (4.7–300 pg/mL). Analytical validation of the newly developed ELISA using mouse serum samples demonstrated good dilutional linearity, good recovery of spiked mouse presepsin, and good reproducibility with intra-assay precision and accuracy.

The digestion sites of CD14 were investigated by Coyne et al., who have suggested that one of the digestion sites is 64–65 of human CD14^15^. These data suggested that CD14 can produce presepsin. We prepared cathepsin D-digested sCD14-His products, and the reactivity to sCD14-His and cathepsin D-digested sCD14-His was determined using the newly developed ELISA. The newly developed ELISA demonstrated low cross reactivity for mouse sCD14-His (the precursor protein); however, the generated digested CD14-His (presepsin) could be detected by the newly developed ELISA (Fig. 4), thereby suggesting its high specificity to mouse presepsin.

The newly developed ELISA revealed that the plasma levels of presepsin were low in healthy mice. The presepsin level was 65.9 ± 21.4 pg/mL; however, the plasma sCD14 level was 43.2 ± 7.2 ng/mL, which was approximately 600 times higher than the presepsin level. Furthermore, the levels of presepsin in the healthy and LPS-injected sepsis models indicated that presepsin is not increased by LPS administration. However, the high levels of sCD14 in the plasma of LPS-injected sepsis mouse model suggested that sCD14 is induced by LPS administration. Endo et al. have revealed no correlation between presepsin and sCD14 in patients with sepsis^16^. This observation suggested that the specificity of ELISA to presepsin is an important performance for determining presepsin in plasma that contains high levels of sCD14.

Models of endotoxemia are of scientific importance in the evaluation of specific biological mechanisms and pathways, such as the immune response to the prototypical stimuli of specific Toll-like receptor pathways, such as LPS and CD14/MD2/TLR4^17^. However, the presepsin levels in the LPS-injected sepsis mouse model were not high in this study compared with the sCD14 levels, suggesting that the induction mechanisms of presepsin and sCD14 are not similar. Presepsin is produced by phagocytosis and through digestion by cathepsin D^4,5^. Therefore, LPS activation may not be sufficient to produce high levels of presepsin. Feng et al. have demonstrated that LPS significantly inhibited phagocytosis of apoptotic neutrophils by mouse peritoneal macrophages, indicating that presepsin production is reduced in the LPS-injected sepsis model^18^. The cecal ligation and puncture (CLP) model is arguably a more physiologically relevant model^19^, and the use of presepsin in CLP models will be investigated in the future. Moreover, a SLE and other mouse models are developed and the newly developed ELISA will be used for investigating the severity of diseases or its production mechanism^20,21^.

In conclusion, the plasma levels of presepsin in mice are low compared with the plasma sCD14 concentrations, and low cross-activity to sCD14 assay is important for the measurement of the presepsin levels. The newly developed ELISA had a good specificity, sensitivity, and precision for determining presepsin and may be a useful tool for evaluating therapeutic interventions in diseases where presepsin is elevated. Moreover, the elevation of presepsin in plasma due to unknown processes related to sCD14 in non-infected cases will be investigated using the newly developed ELISA.

## Methods

The content and execution of the current study were approved by The Ethics regulations of the Center for In Vivo Science Iwate Medical University, Japan (No. 03–020). Authors complied with the ARRIVE guidelines. All methods were carried out in accordance with the guidelines and regulations of The Ethical Committee of the Iwate Medical University.

## Materials

Two synthetic peptides—37-mer peptide (N-pep2: LLVHASPAPPEPCELDEESCSCNFSDPKPDWSSAFNC) and 16-mer peptide (C-pep8: RVDTEADLGQFTDIIK)—were synthesized by Eurofins Genomics (Tokyo, Japan) and used for rabbit immunization. Mouse CD14 protein (His Tag) and mouse cathepsin D (His Tag) were purchased from Sino Biological (Beijing, China). Synthetic 37- and 16-mer peptides were conjugated to keyhole limpet hemocyanin (KLH) (Thermo-Fisher Scientific, Waltham, MA, USA) as the immunogen. ddY mice were obtained from Japan SLC (Shizuoka, Japan). Unless specified otherwise, all chemicals were purchased from Sigma-Aldrich (Tokyo, Japan) or FUJIFILM Wako Pure Chemical Corporation (Osaka, Japan).

### Establishment of anti-mouse presepsin polyclonal antibodies

Presepsin is a 64 amino acid N-terminal fragment of CD14, and N-terminal and C-terminal peptides were selected as immunogens to prepare sandwich ELISA. A peptide immunogen was designed based on the following criteria: (i) it contains the N-terminal of CD14 amino acid sequence; (ii) the amino acid sequence maintains two sulfhydryl bonds; and (iii) the C-terminal of the presepsin amino acid sequence is a homolog of the human presepsin sequence. For the production of the N-terminal antibody, in addition, 5 amino acids of the signal peptide were added as a linker that binds KLH. The 37-mer sequence (N-pep2: LLVHASPAPPEPCELDEESCSCNFSDPKPDWSSAFNC) satisfied the N-terminal sequence criteria (Fig. 5A). We synthesized the 37-mer peptide of mouse presepsin as the immunogen of N-terminal, which had two sulfhydryl bonds and conjugated with KLH at the N-terminal end. For the C-terminal antibody, the mouse presepsin sequence was determined based on the human sequence described in the human presepsin antibody patent (PCT/JP2015/073839). No linker was added for this peptide according to the patent. The 16-mer sequence (C-pep8: RVDTEADLGQFTDIIK) satisfied the C-terminal sequence criteria (Fig. 5B). We synthesized the 16-mer peptide of presepsin and added a Cysteine (Cys) at the N-terminal end for conjugation with KLH as the immunogen.

The immunogens were prepared as follows. For the N-pep2 peptide, KLH was bound to the NH_2_ residue at the N-terminus using Imject EDC mcKLH Spin Kit (Thermo Scientific, Tokyo, Japan) according to the manual. For the C-pep8 peptide, KLH was bound to the Cys-derived SH residue using Imject Maleimide-Activated mcKLH (Thermo Scientific, Tokyo, Japan). Peptide bound KLH were used as an immunogens.

Anti-mouse presepsin polyclonal antibodies were established using immunized KLH-conjugated peptides. Briefly, each two 2–3 kg female New Zealand White rabbits (Japan SLC, Shizuoka, Japan) were injected with 0.3 mg KLH-conjugated synthetic N-pep2 (37-mer) and C-pep8 (16-mer) peptide emulsified in Complete Freund’s Adjuvant (Difco/Becton Dickinson, Franklin Lakes, NJ, USA) via the subcutaneous route. Four additional injections of 0.1 mg KLH-conjugated peptide emulsified in Incomplete Freund’s Adjuvant were administered at 3-week intervals. After the fifth immunization, the rabbits underwent pre-bleeding to determine the antibody titer to immunized peptide and boosted with 0.3 mg adjuvant-free antigen. Seven days after the final injection, blood samples from immunized rabbits were collected aseptically, and the serum was separated.

Rabbit antiserum (N-pep2 or C-pep8) was filtered through a 0.45 μm membrane, and 5 mL (equivalent to 25 mg of IgG) was diluted with 5 mL of phosphate-buffered saline (PBS) (pH 7.4). For example, the method for purifying the N-pep2 antibody was shown below. The diluted antiserum was applied to 5 mL of Protein A resin (Protein A MabSpeed rP202, Mitsubishi Chemical, Tokyo, Japan), followed by washing the column with 100 mL of PBS (pH 7.4). Bound IgG antibodies were eluted with 15 mL of 0.1 M Glycine–HCl (pH 3.0), the eluate was neutralized with 1 M Tris–HCl (pH 8.0), and the solution was concentrated to 5 mL with a 30,000-cut membrane (Amicon Ultra (30 kD, Millipore, Japan). Then, the concentrate was applied to an N-pep2 peptide binding resin column (N-pep2 peptide was coupled to 5 mL of AminoLink Plus Coupling Resin and C-pep8 was coupled to SulfoLink Coupling Resin (Thermo Scientific, Tokyo, Japan) according to the manual), followed by 100 mL of PBS (pH 7.4) washing. The bound peptide-specific IgG antibody was eluted with 0.1 M Glycine–HCl (pH 2.5), and after neutralizing the solution with 1 M Tris–HCl (pH 8.0), the solution was dialyzed against PBS (pH 7.4). The protein concentration was calculated from the absorbance at 280 nm (using a factor of 0.522).

### Preparation of transiently expressed mouse presepsin-Fc

A DNA sequence encoding the extracellular domain (Met 1-Lys 83) of mouse CD14 (UniProtKB -P10810) was fused with the Fc region encoding the CH2 and CH3 domains of the human IgG1 at the C-terminus (Fig. 5A). The DNA sequence encoding mouse presepsin DNA sequence with Fc region was synthesized using restriction enzymes (i.e., EcoRI and BamHI). The fragment obtained by cleaving with restriction enzymes was cloned into an expression plasmid pcDNA6/V5 vector (an expression plasmid for mammalian cells; Thermo-Fisher Scientific,　Tokyo, Japan). Then, the sequence was confirmed and designated as pcDNA-mCD14-ST-Fc.

**Figure 6:**
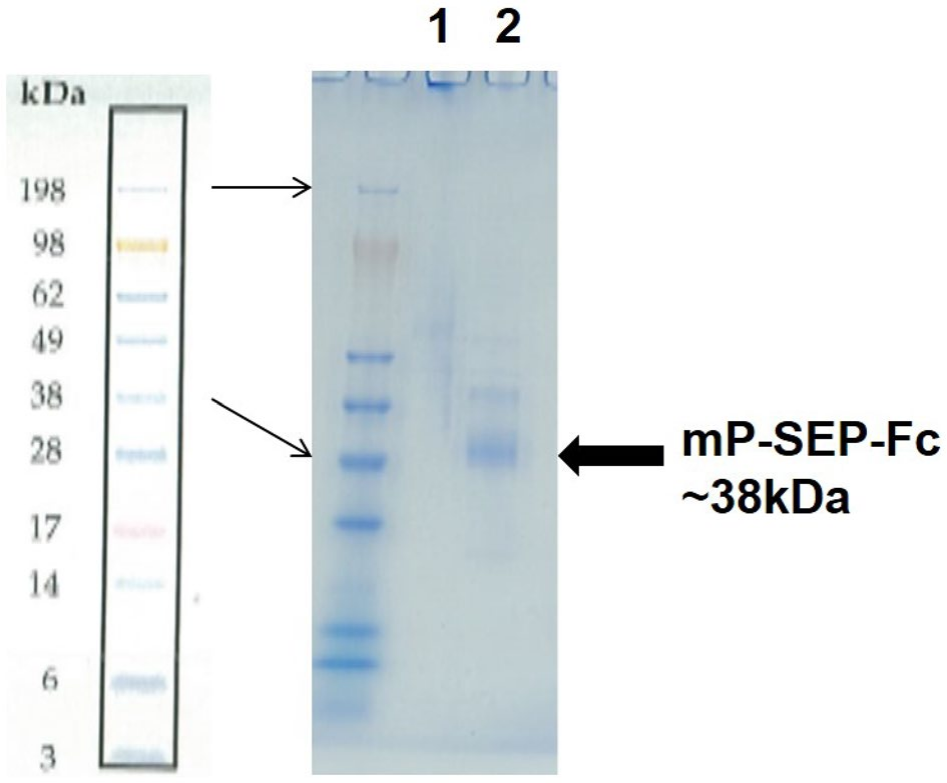
SDS-PAGE of a purified recombinant mouse sCD14-ST-Fc antigen. Purified mouse sCD14-ST-Fc was applied to SDS-PAGE without (1) or with (2) reducing agent and detected by CBB solution. The molecular weight of mouse sCD14-ST-Fc in reduced condition was about 38 kDa (Mouse presepsin; 13 kDa + Human Fc; 25 kDa in reduced condition).

Transfect expression plasmid pcDNA-mCD14-ST-Fc expressing mouse CD14-ST-Fc was transfected into COS-1 cells (ATCC: CRL-1650). That is, 2 μL/mL of the transfection reagent and 4 μg/mL of the plasmid were mixed, added to the medium, added to COS-1 cells, and cultured at 37 °C. After 72 h, the culture supernatant was collected, and a new medium was added. After 96 h, the culture supernatant was collected, mixed, and then filtered through a 0.22 μm filter (Sterivac, Millipore). The obtained culture supernatant was purified using protein A column (Protein A MabSpeed rP202, Mitsubishi Chemical, Tokyo, Japan). The eluate containing the purified product was concentrated and subsequently dialyzed against Dulbecco modified phosphate-buffered saline (D-PBS) (pH 7.4). The protein concentration was determined using the Bradford method with bovine serum albumin (BSA) (BioRad, California, USA) as a standard product. When the obtained rsCD14ST-Fc was analyzed via sodium dodecyl sulfate–polyacrylamide gel electrophoresis under a reduced condition, a single band with a molecular weight of approximately 38 kDa was confirmed (Fig. 6).

Purified mouse rsCD14ST-Fc was used as a provisional standard of mouse presepsin.

### Evaluation of the specific binding of the established antibodies to mouse presepsin

The binding activity of the anti-presepsin antibody to the prepared rsCD14-ST-Fc was confirmed via ELISA, in which mouse sCD14-ST-Fc was immobilized on a solid phase (Fig. 1A). Briefly, a 96-well MaxiSorp plate (Nunc/Thermo-Fisher, Rochester, NY, USA) was coated with mouse sCD14-ST-Fc (1 μg/mL) in PBS (pH 7.4) overnight at 4 °C and then blocked with 0.1% (w/v) BSA/PBS (pH 7.4) for 2 h at room temperature. After the blocking solution was removed, 50 μL of sequential diluted anti-mouse presepsin antiserum (i.e., N-pep2 and C-pep8) by PBS-T (pH7.4) which added 0.1% Tween-20 (FUJIFILM Wako Pure Chemical, Osaka, Japan) was added to each well of the plate. After incubation at 37℃ for 1 h, the plate was washed three times with PBS-T (0.1% Tween-20/PBS), and 50 μL anti-rabbit immunoglobulin horseradish peroxidase (HRP) (Dako, Tokyo, Japan) of 0.1% BSA/PBS-T was added. After washing four times with PBS-T, 50 μL tetramethylbenzidine (TMB) (Sigma-Aldrich, Glostrup, USA) substrate solution was added to each well to initiate the colorimetric reaction. The reaction was stopped by adding 1 M H_2_SO_4_. Then, optical absorbance was measured at 450 nm (630 nm as secondary) using Envision 2102 Multilabel Reader (BioTek, Tokyo, Japan).

The binding activity of the prepared anti-presepsin antibodies was confirmed by ELISA where mouse CD14 His tag was immobilized on a solid phase (Fig. 1B). Briefly, a 96-well MaxiSorp plate was coated with mouse CD14 His tag (1 μg/mL) in PBS (PH 7.4), overnight at 4 °C and then blocked with 0.1% (w/v) BSA/PBS. Then, 50 μL of sequential diluted affinity purified anti-presepsin antibodies was placed into each well of the plate. The plate was incubated at 37 °C for 1 h and washed three times, and 50 μL anti-rabbit immunoglobulin-HRP (Dako, Tokyo, Japan) of 0.1% BSA/PBS-T was added. After washing four times with PBS-T, 50 μL o-phenylenediamine dihydrochloride substrate solution was added, and the reaction was stopped by adding 1 M H_2_SO_4_. Then, optical absorbance was measured at 492 nm.

### Preparation of biotinylated N-pep2 antibody

N-pep2 purified antibody (1–2 mg/mL) dissolved in PBS (pH 7.4) and EZ-Link™ Sulfo-NHS-LC-Biotin (Thermo Scientifics, Tokyo, Japan) dissolved in PBS were mixed at a molar ratio of 1:20 and allowed to stand at room temperature for 30 min. After completion of the reaction, the biotinylated antibody solution was purified with NAP-10 (Cytiva, Tokyo, Japan) equilibrated with 0.1% Tween-20 in Tris-buffered saline (TBS-T) (pH 7.6) to remove free biotin. 1/100 volumes of 10% BSA solution was added, and Proclin-300 (Merck, Darmstadt, Germany) was added to a final concentration of 0.02%, and stored in a refrigerator. The concentration (mg/mL) of biotinylated antibody was taken as the value of antibody divided by the volume (mL).

### Development of a sandwich ELISA specific for mouse presepsin

The C-pep8 antibody selected based on the reactivity and specificity to mouse presepsin was used to coat the plate for developing a sandwich ELISA along with the biotinylated-N-pep2 antibody. Briefly, the antibody was dialyzed against PBS (pH 7.4) and then incubated with EZ-Link^™^ Sulfo-NHS-LC-Biotin (Pierce/Thermo-Fisher, Scientific Rochester, NY, USA) for 30 min at room temperature. Free biotin was removed using NAP-10 column (GE Healthcare, Tokyo, Japan), and buffer was exchanged with 50 mM TBS-T (pH 7.6).

A 96-well MaxiSorp plate was coated with C-pep8 antibody (5 μg/mL) for 1 h at 37℃ and blocked with 0.1% StabilGuard (SurModics, Eden Prairie, MN, USA) and 0.1% Tween-20 for 2 h at room temperature. After washing the plate twice with TBS-T, 50 μL of the samples or standard solution of mouse presepsin (4.7–300 pg/mL) diluted with 2% mouse serum in TBS-T was added and incubated overnight at 4 °C. After washing three times with TBS-T using a plate washer (BioTec Auto-Mini washer AMW-8R), 50 μL biotinylated-N-pep2 antibody solution (0.18 μg/mL in TBS-T) was added and incubated at room temperature for 1 h with shaking (250 rpm). After washing with TBS-T three times, Amdex™ Streptavidin Horseradish Peroxidase Conjugate (Amdex™, RPN4401, Sigma-Aldrich, Glostrup, USA) in TBS-T was added and incubated for 30 min at room temperature. Finally, after washing the plate with TBS-T five times, the reaction was initiated by adding 50 μL ready-to-use TMB solution. Then, 30 min later, the reaction was stopped using 1 M H_2_SO_4_ solution, and an absorbance of 450–630 nm of each well was measured using an TECAN Sunrise Microplate Reader (Tecan, Hännedorf, Switzerland).

### Determination of CD14-His and cathepsin D-digested CD14-His using the developed ELISA

Mouse presepsin was prepared in-house by cleavage of the recombinant CD14-His protein by mouse cathepsin D. Briefly, mouse CD14-His (100 μg) was reconstituted using a digestion buffer (0.1 M glycine–HCl (pH 3.5), 0.1% Tween-20, and 0.15 M NaCl) to obtain 1 μg/μL solution. The pH was adjusted to 3.5 using 1 M HCl. Then, 3.33 μg mouse cathepsin D was added and incubated for 1 h at 37 °C. The reaction was stopped, and the buffer was changed to 0.1% Tween-20 TBS (pH 7.6). Subsequently, the reaction solution was analyzed using presepsin ELISA.

### Comparison of mouse presepsin and sCD14 levels between healthy mice and an LPS-injected mouse sepsis model

Blood samples were collected from the tail vein using a heparinized syringe to determine the plasma levels of intact presepsin and sCD14 in 6-week-old male healthy ddY mice (Japan SLC, Shizuoka, Japan). Twenty blood samples were centrifuged at 2000 g for 15 min at 4 °C, and plasma fractions were collected and used to determine the levels of presepsin and sCD14 using the developed mouse presepsin kit and the commercially available Quantikine CD14 ELISA kit (R&D systems, Minneapolis, MN, USA). Data were expressed as means ± standard deviations.

The same 20 male ddY mice were used to develop an LPS-injected mouse sepsis model. Then, 2 μg/g body weight of *E. coli* O111 LPS (Sigma-Aldrich, St. Louis, MO) was intraperitoneally injected to establish a septic model^22^. The mice were bled 4 h after LPS challenge under isoflurane anesthesia from cardiocentesis to measure the plasma presepsin and sCD14 concentrations.

The concentrations of mouse presepsin were determined. The Manne-Whitney U test was used to compare presepsin and sCD14 levels between the normal mouse and LPS-injected mouse. The calculations we performed with the statistical software StatFlex version 6.0 (Artec Co., Ltd. Oosaka, Japan). P values lower than 0.05 were considered significant.

## Supplementary Information

Supplementary Information.

## Data Availability

The datasets used and/or analysed during the current study available from the corresponding author on reasonable request.
