# Supplementary material for: Development of a newly immunoassay specific for mouse presepsin (sCD14-ST)
Source: Sci Rep. 2022 Dec 15;12:21724. doi: 10.1038/s41598-022-22096-1 (PMC9755121; doi:10.1038/s41598-022-22096-1)
Supplement: Supplementary file 1 — Supplementary Information. [file 41598_2022_22096_MOESM1_ESM.docx]

Supplementary information


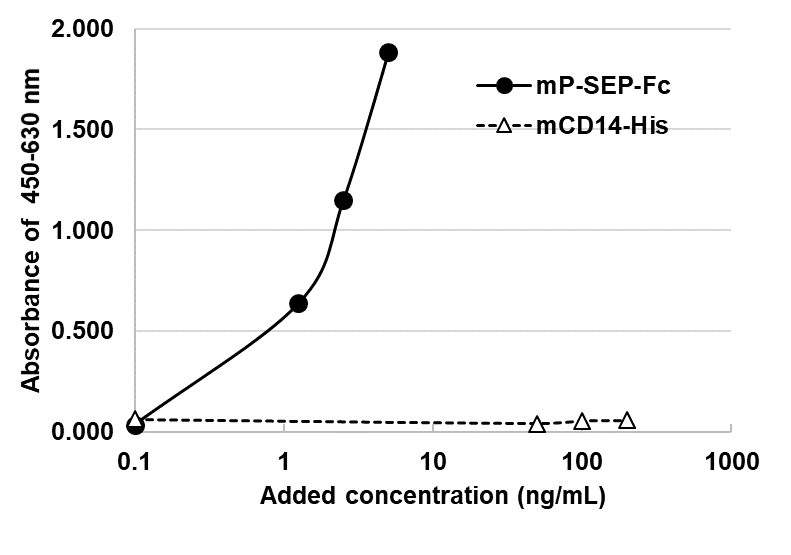


Figure 7 Cross reactivity of mouse CD14 （mCD14-His) in mouse presepsin sandwich ELISA.

Recombinant mouse CD14-His was diluted to 50, 100, and 200 ng/mL and added to the presepsin ELISA kit and determined the cross-reactivity.

Figure 8A Western Blotting analysis of mouse CD14-His and cathepsin D digested CD14-His.

1 2

1 2

1 2

1 2

KDa

49 →

17→

←CD14-His

←Digested CD14-His

（Presepsin）

1：mouse CD14-His

2：Digested mouse CD14-His


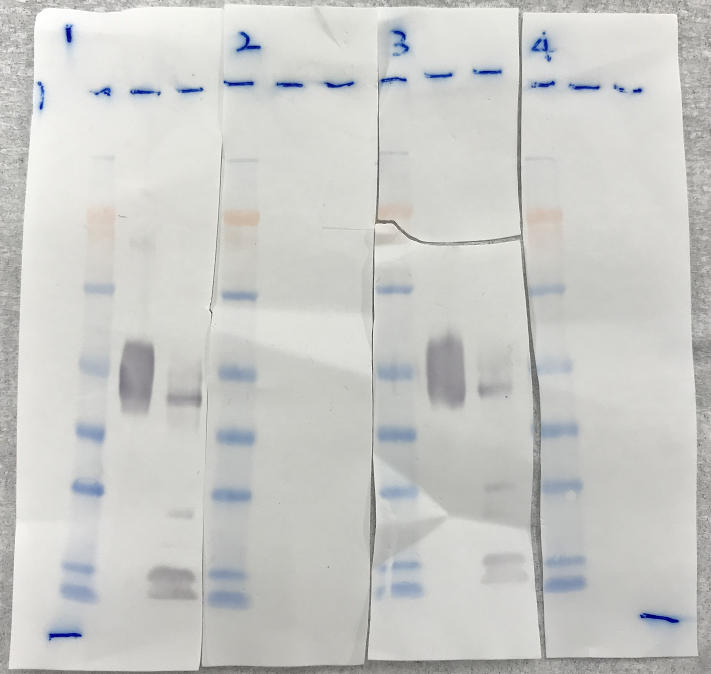


Anti-His Ab

N.C.

(-Ab)

Anti-N-pep2 Ab

Anti-C-pep8 Ab


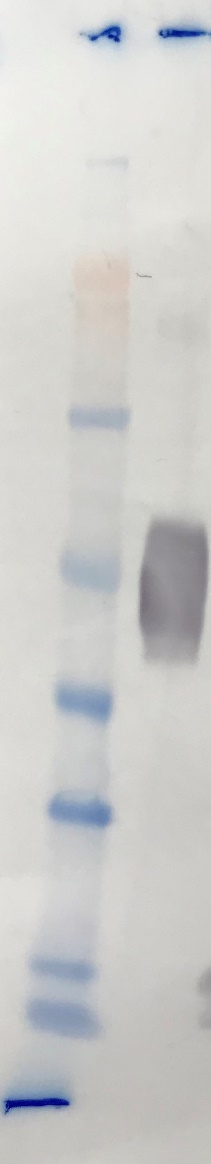

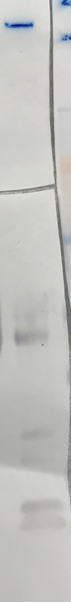


1 2

KDa

49 →

17→

←CD14-His

←Digested CD14-His

（Presepsin）

1：mouse CD14-His/Anti-His Ab

2：Digested mouse CD14-His/Anti-N-pep2 Ab

Figure 8B Western Blotting analysis of mouse CD14-His and cathepsin D digested CD14-His.
